# Identification of a pathogenic *SMCHD1* variant in a Chinese patient with bosma arhinia microphthalmia syndrome: a case report

**DOI:** 10.1186/s12920-024-01907-6

**Published:** 2024-05-21

**Authors:** Jun-Lin Yang, Heng Gu, Zhuang-Zhuang Yuan, Xiao-Hui Xie, Yi-Feng Yang, Zhi-Ping Tan

**Affiliations:** 1 Department of Cardiovascular Surgery, the Second Xiangya Hospital of Central South University, Changsha, China; 2 Clinical Center for Gene Diagnosis and Therapy, Department of Cardiovascular Surgery, The Second Xiangya Hospital of Central South University, Changsha, Hunan 410011 China; 3 Department of Cell Biology, School of Life Sciences, Central South University, Changsha, China

**Keywords:** Bosma arhinia microphthalmia syndrome, *SMCHD1*, Whole-exome sequencing, Arhinia

## Abstract

**Background:**

Bosma arhinia microphthalmia syndrome (BAMS; MIM603457) is a rare genetic disorder, predominantly autosomal dominant. It is a multi-system developmental disorder characterized by severe hypoplasia of the nose and eyes, and reproductive system defects. BAMS is extremely rare in the world and no cases have been reported in Chinese population so far. Pathogenic variants in the *SMCHD1* gene (MIM614982) cause BAMS, while the underlying molecular mechanisms requires further investigation.

**Case presentation:**

In this study, a Chinese girl who has suffered from congenital absence of nose and microphthalmia was enrolled and subsequently submitted to a comprehensive clinical and genetic evaluation. Whole-exome sequencing (WES) was employed to identify the genetic entity of thisgirl. A heterozygous pathogenic variant, NM_015295, c.1025G > C; p. (Trp342Ser) of *SMCHD1* was identified. By performing very detailed physical and genetic examinations, the patient was diagnosed as BAMS.

**Conclusion:**

This report is the first description of a variant in *SMCHD1* in a Chinese patient affected with BAMS.Our study not only furnished valuable genetic data for counseling of BAMS, but also confirmed the diagnosis of BAMS, which may help the management and prognosis for this patient.

**Supplementary Information:**

The online version contains supplementary material available at 10.1186/s12920-024-01907-6.

## Introduction

Arhinia, a congenital anomaly characterized by the total absence of the nose, is an exceedingly rare malformation with fewer than 100 cases reported to date [1]. This malformation can manifest as an isolated condition or may be accompanied by ocular defects and hypogonadotropic hypogonadism, which together form a potentially life-threatening triad known as Bosma arhinia microphthalmia syndrome (BAMS; MIM603457) [2]. BAMS is a rare genetic disorder, predominantly autosomal dominant. Arhinia is believed to arise from the failure of fusion between the maxillary and lateral nasal processes and the associated abnormal fusion of the cribriform plate during embryonic development [3]. Although the pathogenesis of this condition is presumed to be genetic, the etiology of this severe abnormality remains unknown.

Structural Maintenance of Chromosomes Flexible Hinge Domain Containing 1 (*SMCHD1*, MIM614982), located in chromosome 18p11.32, encodes a 2005 amino acid protein. SMCHD1 is an atypical member of the SMC protein family, containing a C-terminal SMC hinge domain and an N-terminal ATPase domain [4–6]. SMCHD1 was previously shown to act as an epigenetic regulator of autosomal and X-linked genes that plays critical roles during development [7, 8]. In situ hybridization data has indicated regional expression of Smchd1 in the nasal cavity in E14.5 mice, and transcriptional profiling of mouse postnatal olfactory epithelium has revealed that Smchd1 is specifically expressed in immature olfactory sensory neurons [9, 10].

*SMCHD1* function is highly relevant to human disease, including BAMS and facioscapulohumeral muscular dystrophy type 2 (FSHD2; MIM158901) [11]. Through a combination of whole-exome, whole-genome and targeted sequencing in an international cohort of 40 arhinia patients, Shaw et al. discovered a high prevalence (84%) of missense variants in the gene *SMCHD1* [1, 10]. Notably, truncation variants of *SMCHD1* have been found to be common in FSHD2, a rare, oligogenic form of muscular dystrophy [1, 12]. Nevertheless, little is currently known about the genes responsible for causing BAMS or the molecular mechanisms by which *SMCHD1* achieves its various functions.

Here, we reported the first case with BAMS in Chinese population. WES and Sanger sequencing were applied to identify the pathogenic genes of this girl.

## Case presentation

### Clinical manifestations

**Fig. 1:**
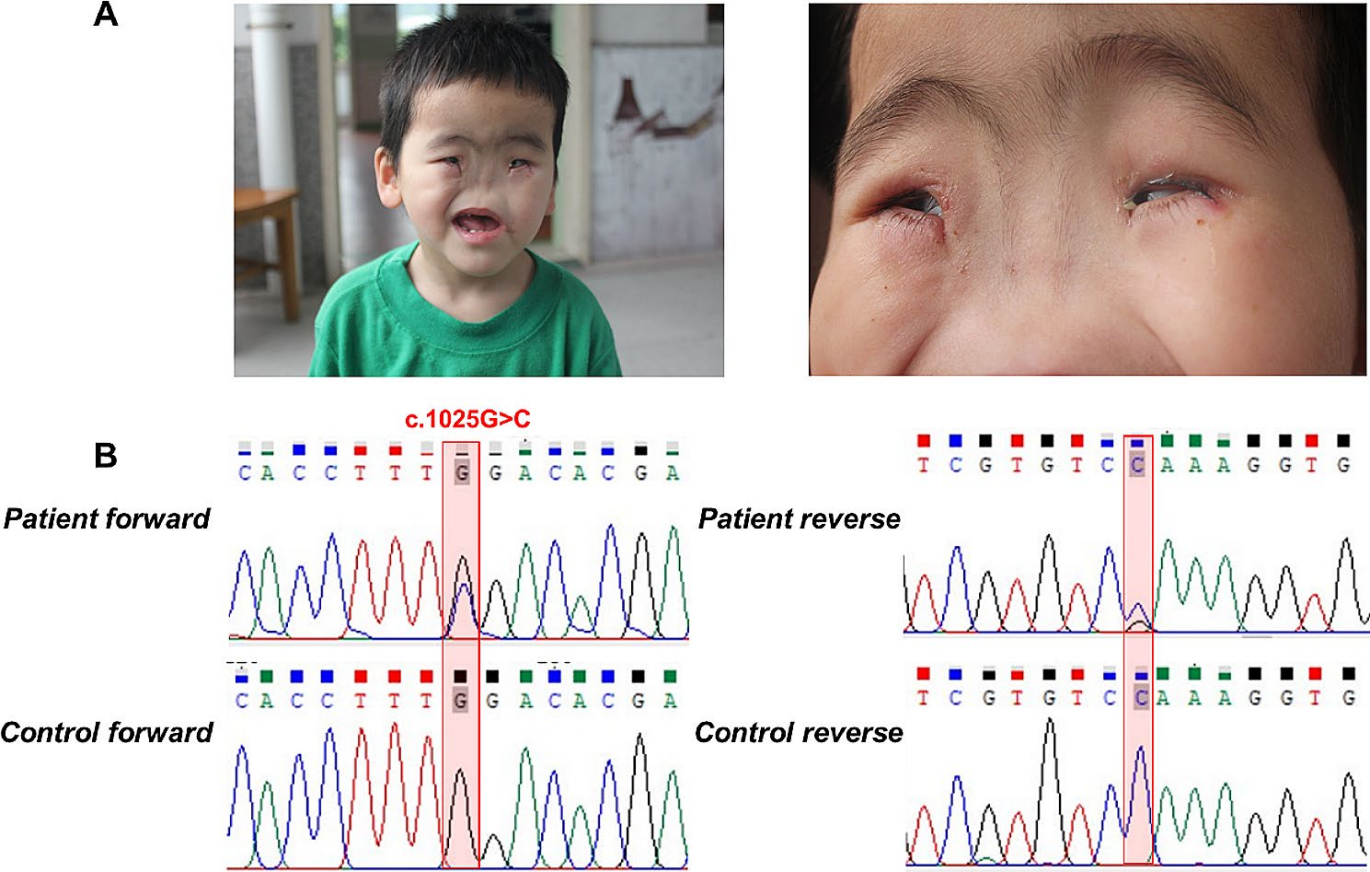
The clinical and sequencing data of this patient. (**A**) Clinical features of this patient, including complete absence of nose and microphthalmia. Consent was obtained to publish patient images. (**B**) Sanger DNA sequencing chromatogram detected a heterozygous missense variant (NM_015295, c.1025G > C; p.Trp342Ser) of *SMCHD1* gene in the patient

The patient, a 9-year-old girl, was born with congenital arhinia and raised at Guangzhou City Social Welfare Institute. Her physical and intellectual development was similar to that of normal peers but was too young to be sure of hypogonadotropic hypogonadism (Table 1). However, the combination of congenital nasal deformities and microphthalmia in this patient suggested a diagnosis of BAMS (Fig. 1A). Moreover, by performing a very detailed physical examination, it was determined that her visual refraction muscle strength and tone were all functioning normally. Unfortunately, we could not exclude a later onset of a muscle phenotype as first signs of FSHD are usually only visible at the end of the second decade of life. However, an MRI examination was not conducted due to the patient’s preferences. Considering that the patient had no signs of muscular dystrophy, the diagnosis of FSHD2 was ruled out for now. Based on these findings, it was concluded that the patient was suffering from BAMS.

**Table 1:** Phenotypic Features of the patient with BAMS

Gender	Age	Consanguinity	Nose	Eyes	Reproductive system	Growth	Psychomotor development
Female	9 Y	Unknown	Complete arhinia	Microphthalmia; Normal eyesight	Normal	Normal	Normal

### Genetic analysis

**Fig. 2:**
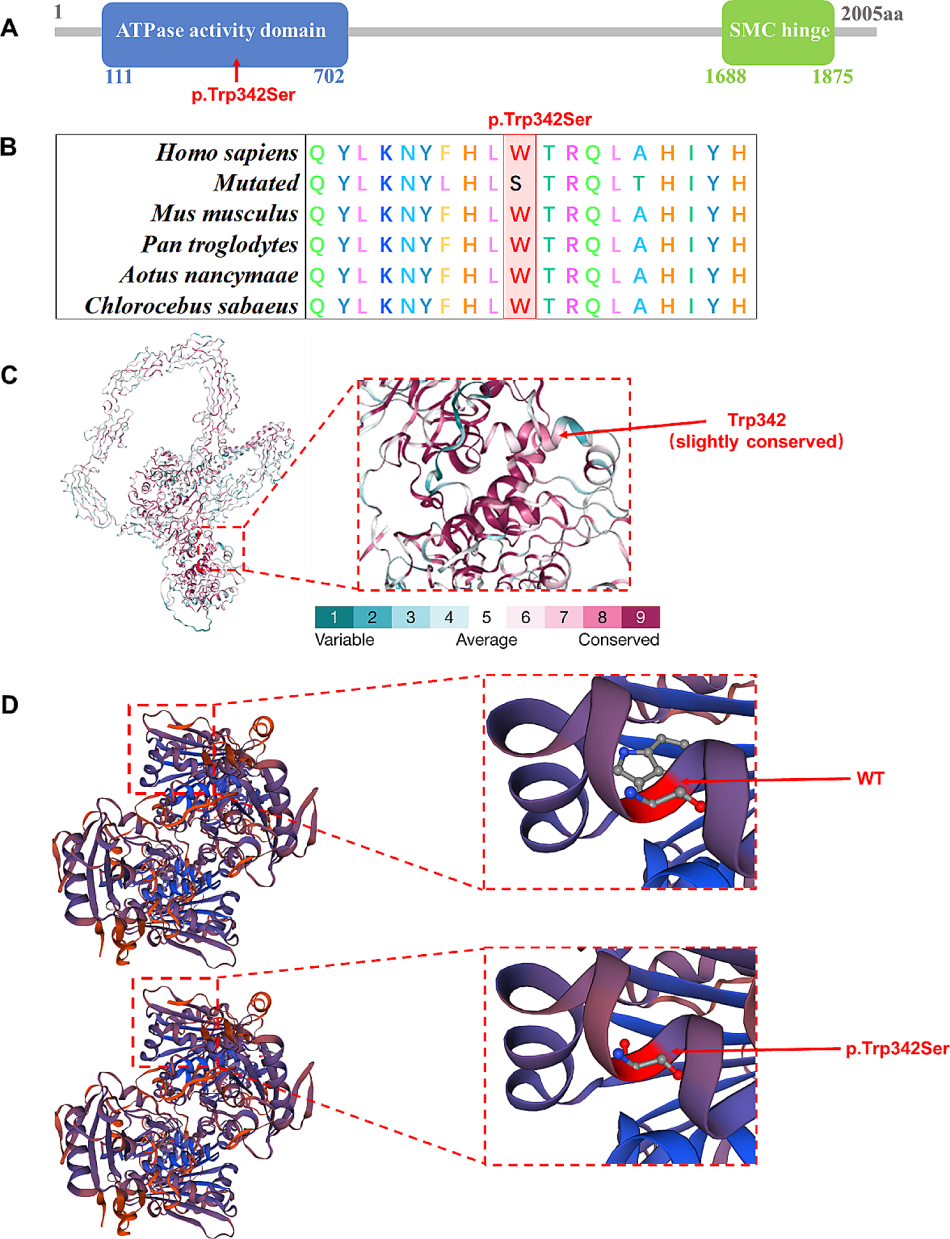
The bioinformatics analysis of this variant. (**A**) The positions of this missense variant in SMCHD1 identified in the patient. Domains in SMCHD1 are indicated with different colored squares. (**B**) Alignment of multiple SMCHD1 protein sequences across species. Letters in red show the W342 site is evolutionarily conserved. (**C**) The conservation analysis of the W342 site amino acids was predicted by ConSurf Server software. (**D**) Structure prediction of wild type and mutant SMCHD1 protein. The wild type SMCHD1 (SMCHD1-WT) protein structure and the p.Trp342Ser mutant SMCHD1 (SMCHD1-p.Trp342Ser) protein structure were predicted by SWISS-MODEL online software

Genomic DNA was extracted from the patient by QIAamp DNA Blood Mini Kit (250) (Qiagen, Valencia, CA, U.S.A). However, parental DNA of the patient were not available due to the unknown parentage. The WES analysis was mainly conducted in the Novogene Bioinformatics Institute (Beijing, China). All variants were formatted under HGVS nomenclature [13]. After data filtration (supplementary material), a pathogenic heterozygous variant of *SMCHD1*, NC_000018.10:g.2694678G > C, NM_015295.3:c.1025G > C, NP_056110.2:p.(Trp342Ser), was identified. Sanger sequencing was further performed to validate this variant (Fig. 1B). This variant results in a change in the amino acid sequence of the ATPase active structural domain of the SMCHD1 protein, which may lead to altered ATPase activity and hence affect the characteristics of the resulting protein. It was predicted to be “disease causing” by MutationTaster, SIFT and PolyPhen2, and also was not found in the 1,000 Genome Browser, The ExAC Browser, the Exome Variant Server and GnomAD. According to ACMG standards and guidelines [14], this variant was categorized as pathogenic (PM1, PM2, PS3, PP3, PP5) (Table 2). Although this variant was reported in a previous research, the pathogenic analysis was absent [10]. We further performed bioinformatics analysis of the variant. Alignment of SMCHD1 amino acid sequences was highly conserved across species (Fig. 2B). Also, ConSurfServer software predicted that the affected amino acid was slightly conserved (Fig. 2C). Furthermore, there reveals a difference between the normal and mutant protein models constructed with SWISS-MODEL software, which affect highly conserved residues and hence affect the SMCHD1 protein features (Fig. 2D). Considering the clinical phenotypes and genetic results, the patient was diagnosed as BAMS.

**Table 2:** The SMCHD1 variant identified by WES for the affected individual

Gene	CHR	RefSeq ID	AA Alteration	Genotype	Function	Database	MutationTaster	CADD	ACMG
SMCHD1	18p11.32	NM_015295	c.1025G > C; p.Trp342Ser	Het	Missense	Unknown variant	Disease causing	6.708873, 32	PM1 + PM2 + PS3 + PP3 + PP5 (Pathogenic)

CHR Chromosome, AA Amino Acids, Het Heterozygous, PVS Pathogenicity very strong, PM Pathogenicity moderate. The database included 1000G, ExAC and Exome Variant Server

## Discussion

In this study, we reported a Chinese girl who has suffered from congenital arhinia and microphthalmia. The patient was adopted by Guangzhou City Social Welfare Institute so that the genomic information of her parents is unknown. WES was conducted to identify the causative genes of this patient. A pathigenic heterozygous missense variant of *SMCHD1*, NM_015295: c.1025G > C: p. (Trp342Ser) was identified in the patient. Sanger sequencing subsequently confirmed this variant. Thus, the patient was further diagnosed as BAMS. Our study further confirms that variants of *SMCHD1* are associated with BAMS.

Consistent with prior researches, the p.(Trp342Ser) variant identified in this study is located in the ATPase activity domain of SMCHD1 protein. As shown in the Fig. 2A, variations in the affected residue of the SMCHD1 protein may lead to different alterations and subsequently impact its function. Although the underlying pathogenesis necessitates further investigation, a detailed functional analysis such as testing of the methylation level of the D4Z4 repeat as done for other BAMS-associated mutations, of the SMCHD1 protein with this heterozygous missense variant is recommended. Such an analysis may provide additional insights into the pathogenic mechanism of BAMS.

There is a lack of clarity on the cause of the different clinical outcomes of pathogenic *SMCHD1* variants. Although previous studies have highlighted the involvement of these pathogenic *SMCHD1* variants in FSHD2, recent reports have also implicated them in the pathogenesis of BAMS [1, 10]. To the best of our knowledge, no individual afflicted with BAMS has yet to exhibit clinical characteristics reminiscent of FSHD2. It has been reported that missense variants in *SMCHD1* were considerably prevalent in BAMS cases, while loss of function variants have been more frequently associated with the manifestation of FSHD2. As reported in published literature, all BAMS-related variants were missense alleles, localized to exons 3–13 of *SMCHD1*, which encodes the ATPase domain of SMCHD1 [1, 10, 15]. And yet in FSHD2, missense, nonsense, and deletion variants spanned the entire *SMCHD1* coding region [1, 11, 16]. Therefore, despite the overwhelming evidence that BAMS is caused by gain-of-function variants in *SMCHD1*, the loss-of-function versus gain-of-function dichotomy between FSHD2 and BAMS appears to be one-sided. It is more likely that both BAMS and FSHD2 are triggered by complex oligogenic or multifactorial mechanisms that only partially intersect at the level of *SMCHD1* [16, 17]. This highlights the need to probe the molecular mechanisms underlying how variations within the same gene can give rise to distinct phenotypic manifestations. Furthermore, A previous study has proposed that the localisation of missense variants within the ATPase structural domain of SMCHD1 may account for the disparate phenotypic outcomes observed in BAMS and FSHD2 cases [18]. However, to fully decipher the impact of *SMCHD1* variants on its function, further studies incorporating structural and biochemical characterizations are warranted.

BAMS is a clinically heterogeneous disease, with a phenotypic spectrum spanning from the absence of craniofacial features to nasal hypoplasia and complete arhinia, rendering clinical diagnosis a challenging task. The findings in Xenopus model indicated that variants implicated in BAMS are associated with a reduced eye diameter, and in severe cases, anophthalmia may ensue [10, 15]. By identifying the relevant cell type (cranial placode) and mechanism of cell death (DUX4), Kaoru et al. proposed that in patients with arhinia and related nasal phenotypes (e.g., anosmia and nasal hypoplasia), nasal morphogenesis is completely or partially arrested when *SMCHD1* missense mutations unleash DUX4 toxicity in cranial placode cells, leading to cell death [19]. Those findings suggested that SMCHD1 plays an important role in the development of craniofacial organs.

In conclusion, we used WES to explore the genetic entity in a Chinese girl who has suffered from congenital absence of nose and microphthalmia. A heterozygous missense variant, NM_015295:c.1025G > C:p.(Trp342Ser), of *SMCHD1* was identified in the patient with BAMS. Here we reported the first case with BAMS in Chinese population. Our investigation not only offers crucial genetic counseling data to the affected individual, but also furnishes characteristic clinical images of BAMS, which can aid in the accurate diagnosis of the disease in conjunction with genetic analyses.

### Electronic supplementary material

Below is the link to the electronic supplementary material.

Supplementary Material 1

## Data Availability

The datasets used during the current study available from the corresponding author on reasonable request.

## Abbreviations

BAMS: Bosma arhinia microphthalmia syndrome
WES: Whole Exome Sequencing
SMCHD1: Structural Maintenance of Chromosomes Flexible Hinge Domain Containing 1
FSHD2: Facioscapulohumeral muscular dystrophy type 2
ACMG: American College of Medical Genetics and Genomics
PVS: Pathogenicity very strong
PM: Pathogenicity moderate
1000G: The 1000 Genomes Project

## References

[1] Shaw ND, Brand H, Kupchinsky ZA, Bengani H, Plummer L, Jones TI (2017). SMCHD1 mutations associated with a rare muscular dystrophy can also cause isolated arhinia and Bosma arhinia microphthalmia syndrome. Nat Genet.

[2] Brasseur B, Martin CM, Cayci Z, Burmeister L, Schimmenti LA (2016). Bosma arhinia microphthalmia syndrome: clinical report and review of the literature. Am J Med Genet A.

[3] Graham JM, Lee J (2006). Bosma arhinia microphthalmia syndrome. Am J Med Genet A.

[4] Chen K, Czabotar PE, Blewitt ME, Murphy JM (2016). The hinge domain of the epigenetic repressor Smchd1 adopts an unconventional homodimeric configuration. Biochem J.

[5] Chen K, Dobson RC, Lucet IS, Young SN, Pearce FG, Blewitt ME (2016). The epigenetic regulator Smchd1 contains a functional GHKL-type ATPase domain. Biochem J.

[6] Chen K, Hu J, Moore DL, Liu R, Kessans SA, Breslin K (2015). Genome-wide binding and mechanistic analyses of Smchd1-mediated epigenetic regulation. Proc Natl Acad Sci U S A.

[7] Blewitt ME, Gendrel AV, Pang Z, Sparrow DB, Whitelaw N, Craig JM (2008). SmcHD1, containing a structural-maintenance-of-chromosomes hinge domain, has a critical role in X inactivation. Nat Genet.

[8] Mould AW, Pang Z, Pakusch M, Tonks ID, Stark M, Carrie D (2013). Smchd1 regulates a subset of autosomal genes subject to monoallelic expression in addition to being critical for X inactivation. Epigenetics Chromatin.

[9] Nickell MD, Breheny P, Stromberg AJ, McClintock TS (2012). Genomics of mature and immature olfactory sensory neurons. J Comp Neurol.

[10] Gordon CT, Xue S, Yigit G, Filali H, Chen K, Rosin N (2017). De novo mutations in SMCHD1 cause Bosma arhinia microphthalmia syndrome and abrogate nasal development. Nat Genet.

[11] Lemmers RJ, Tawil R, Petek LM, Balog J, Block GJ, Santen GW (2012). Digenic inheritance of an SMCHD1 mutation and an FSHD-permissive D4Z4 allele causes facioscapulohumeral muscular dystrophy type 2. Nat Genet.

[12] Qiu LL, Lin XD, Xu GR, Wang LL, Ye ZX, Lin F (2021). A novel start codon variant in SMCHD1 from a Chinese family causes facioscapulohumeral muscular dystrophy type 2. Chin Med J (Engl).

[13] den Dunnen JT, Dalgleish R, Maglott DR, Hart RK, Greenblatt MS, McGowan-Jordan J (2016). HGVS recommendations for the description of sequence variants: 2016 update. Hum Mutat.

[14] Richards S, Aziz N, Bale S, Bick D, Das S, Gastier-Foster J (2015). Standards and guidelines for the interpretation of sequence variants: a joint consensus recommendation of the American College of Medical Genetics and Genomics and the Association for Molecular Pathology. Genet Med.

[15] Gurzau AD, Chen K, Xue S, Dai W, Lucet IS, Ly TTN (2018). FSHD2- and BAMS-associated mutations confer opposing effects on SMCHD1 function. J Biol Chem.

[16] Jansz N, Chen K, Murphy JM, Blewitt ME (2017). The Epigenetic Regulator SMCHD1 in Development and Disease. Trends Genet.

[17] Mul K, Lemmers R, Kriek M, van der Vliet PJ, van den Boogaard ML, Badrising UA (2018). FSHD type 2 and Bosma arhinia microphthalmia syndrome: two faces of the same mutation. Neurology.

[18] Lemmers R, van der Stoep N, Vliet PJV, Moore SA, San Leon Granado D, Johnson K (2019). SMCHD1 mutation spectrum for facioscapulohumeral muscular dystrophy type 2 (FSHD2) and Bosma arhinia microphthalmia syndrome (BAMS) reveals disease-specific localisation of variants in the ATPase domain. J Med Genet.

[19] Inoue K, Bostan H, Browne MR, Bevis OF, Bortner CD, Moore SA (2023). DUX4 double whammy: the transcription factor that causes a rare muscular dystrophy also kills the precursors of the human nose. Sci Adv.

