# Supplementary figures and images for: Identification of a pathogenic SMCHD1 variant in a Chinese patient with bosma arhinia microphthalmia syndrome: a case report
Source: BMC Med Genomics. 2024 May 21;17:136. doi: 10.1186/s12920-024-01907-6 (PMC11110391; doi:10.1186/s12920-024-01907-6)

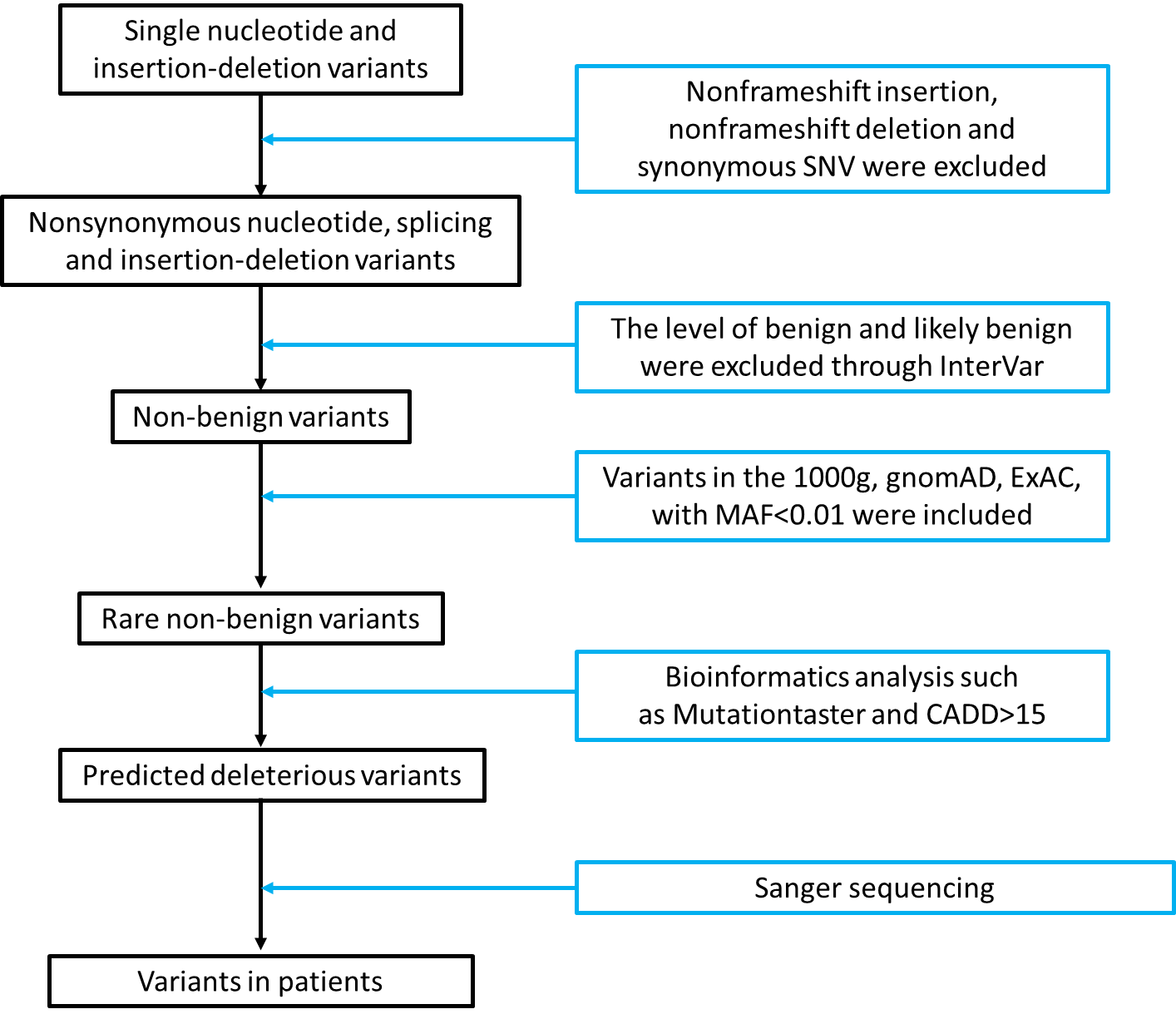
**Supplementary Figure. WES data filtering and validation procedure.**

Supplement: Supplementary file 1 — Supplementary Material 1 [file 12920_2024_1907_MOESM1_ESM.docx]
